# Preparation of 2D ZIF-L and Its Antibacterial and Antifouling Properties

**DOI:** 10.3390/nano13010202

**Published:** 2023-01-02

**Authors:** Jingyu Li, Yang Zhang, Haichao Zhao, Guoxin Sui

**Affiliations:** 1 Shi-Changxu Innovation Center for Advanced Materials, Institute of Metal Research, Chinese Academy of Sciences, Shenyang 110016, China; 2 School of Materials Science and Engineering, University of Science and Technology of China, Shenyang 110016, China; 3 Key Laboratory of Marine Materials and Related Technologies, Zhejiang Key Laboratory of Marine Materials and Protective Technologies, Ningbo Institute of Materials Technology and Engineering, Chinese Academy of Sciences, Ningbo 315201, China

**Keywords:** ZIF-L, two dimensional, antibacterial, anti-algae

## Abstract

The excessively leached metal ions from traditional metallic antimicrobial nanoparticles are harmful to biological and human tissues. Metal-organic frameworks (MOFs) coordinating bioactive metal ions to organic bridging ligands can potentially address this issue, avoiding the excessive leaching of metal ions and simultaneously exhibiting high effective antibacterial activities. Here, we report the preparation of a 2-dimensional leaves-like zeolitic imidazolate framework (ZIF-L) for potential antibacterial and anti-algae applications. The ZIF-L nanosheet exhibits complete inactivation of *Escherichia coli* (phosphate buffer saline: 4 h) and *Bacillus subtilis* (seawater: 0.5 h). The ZIF-L/epoxy composite has excellent antibacterial effect, poisoning effect and anti-adhesion effect on a variety of marine algae. It is worth noting that the removal rate (*Escherichia coli*) for ZIF/epoxy composite can be reached to 90.20% by only adding ZIF-L (0.25 wt%). This work will inspire researchers to develop more metal-organic frameworks materials for applications in the antibacterial and anti-algae fields.

## 1. Introduction

The adhesion and growth of marine microorganisms on the surface of facilities result in marine biofouling and pollution, which has a profound impact on the marine economy [1,2]. At present, the development of effective protection methods for marine facilities has received more and more attention [3]. Anti-fouling biocides [4] have been commonly used as additives to protect submerged structures from the undesirable adhesion of fouling organisms. Before organotin compounds (such as tributyltin) are used to control fouling organisms in the 1960s [5], a variety of toxic materials (such as copper [6], mercury [7], arsenic [8] are widely used. However, compounds based on tributyltin may lead to harmful damage to the marine environment, which have been banned by countries since 2008 [5]. Antibiotics can selectively kill bacteria, but they have no broad-spectrum bactericidal properties and overuse of antibiotics will produce resistant strains of super bacteria. Therefore, it is urgent to develop alternative ways to kill bacteria, preventing bacteria attachment and biofouling.

Nanomaterials are one of the most promising materials [9] for antibacterial and antifouling application because of their unique physical and chemical characteristics [10], which are rarely expressed in their bulk form [11]. In terms of unique chemical and physical properties, two-dimensional (2D) nanomaterials [12], including graphene [13,14], graphitic carbon nitride [15,16], metal oxides [17], transition-metal oxides [18], transition-metal dichalcogenides [19], and layered double hydroxides [20] have attracted great research interest. Xu has successfully developed the graphene oxide/TiO_2_-polyvinylidene fluoride hybrid ultrafiltration membrane with high BSA rejection rate (92.5%) [21]. Maxine [22] has prepared graphene-silver nanocomposites (antibacterial properties) using load silver nanoparticles on graphene sheets, which can effectively inhibit the growth of organisms. Sun [23] has demonstrated a model containing sodium paeonolsilate intercalated Zn_2_Al layered double hydroxides and acrylic resin with a slower release rate of PAS (0.54 ppm/d).

ZIF-8 [24] is one kind of MOFs material with a zeolite skeleton structure, consisting of inorganic ligand-transition metal ion and organic ligand-imidazole or imidazole derivative. Chen [25] has reported that a 2D zeolite imidazolate framework material is synthesized by adjusting the ratio of Hmim/Zn ratio in the aqueous solution. Ding [26] has synthesized the zeolitic imidazate framework material (ZIF-L) with hierarchical morphology by hydrothermal method. Xia [27] has obtained the defect-rich graphene nanomesh of 2D metal-organic frameworks for the oxygen reduction reaction. The antifouling material and antibacterial properties [28,29] based on ZIF-8 or ZIF-8 derived nanoparticles have been studied by a few scientists. Halim [30] has developed nylon 6,6 nanofiber membrane, incorporating zeolitic imidazolate framework-8 (ZIF-8) as the additive for water filtration and give more than 80% rejection of oil. Zhang [31] has reported that the positively-charged ZIF-L nano-daggers could impose a strong electrostatic interaction selectively to more negatively-charged microbial cell membranes. ZIF-L coated onto cotton gauze could be used to handle infected wounds in mice because of ZIF-L nano-array surfaces killing bacteria efficiently via physical interaction [32].

Herein, the ZIF-L composite is designed with a 2-dimensional leave-like morphology through precise control of reactant concentration and reaction time by simple aqueous solution synthesis method. ZIF-L have high antibacterial activity in simulate physiological environment and marine environment by releasing Zn^2+^ in various pH solutions. The ZIF-L/epoxy composite (epoxy as matrix resins) has an excellent antibacterial effect (Enterobacteria *Escherichia coli* and marine bacteria *Bacillus subtilis*) and an anti-adhesion effect on a variety of marine algae. This work will inspire researchers to develop much more advanced applications of metal-organic materials in medical devices, tools and implants. 

## 2. Materials and Methods

### 2.1. Materials

Zinc nitrate hexahydrate (Zn(NO_3_)_2_·6H_2_O), ethanol and methanol were obtained from Sinopharm Chemical Reagent Co., Ltd., (Shanghai, China). In addition, 2-Methylimidazole (2-MeIM) and Poly(propylene glycol) bis(2-aminopropyl ether) (D230) were purchased from Aladdin., (Shanghai, China). Calcein-AM/PI Double Stain Kit 40747ES76 was obtained from Shanghai Yeasen Biotechnology Co., Ltd., (Shanghai, China). Centrifuge tubes, sterile culture dishes, pipettes and pipette tips, sterile well plates, semipermeable membrane, etc. were purchased from Ningbo Zhenhai Hangjing Biotechnology Co., Ltd., (Ningbo, China). Nutrient agar, LB broth, 2216E liquid medium and 2216E agar were purchased from Qingdao Hope Bio-Technology Co., Ltd., (Qingdao, China). *Escherichia coli* (ATCC 25922) and *Bacillus subtilis* ATCC6633 were provided by Shanghai Luwei Technology Co., Ltd., (Shanghai, China). Waterborne epoxy resin was obtained from Ningbo Institute of Materials Technology and Engineering, Chinese Academy of Sciences, (Ningbo, China). *Phaeodactylum tricornutum*, *Chlorella* and *Navicula torgutum* were purchased from Yueqing Guangyu Biological Technology Co., Ltd., (Shanghai, China). Glutaric dialdehyde solution (for electron microscope, 2.5%) was supplied by Huayueyang Biotechnology Co., Ltd., (Beijing, China). All other chemicals and reagents were of analytical grade and used as received.

### 2.2. Synthesis of ZIF-L

**Scheme 1:**
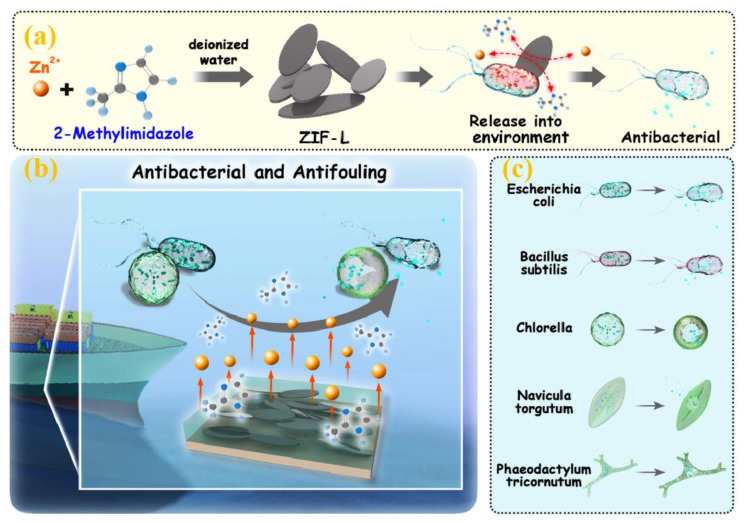
Schematic Illustration of (**a**) the preparation of ZIF-L and antibacterial mechanism, (**b**) antifouling and antibacterial effects of ZIF-L-based composites against algae and bacteria, (**c**) the morphology changes of bacteria and algae after being treated by ZIF-L.

The procedure for synthesis of ZIF-L with leaf-like morphology was presented in Scheme 1. Typically, Zn(NO_3_)_2_·6H_2_O (0.4 g) and 2-MeIM (1.1 g) were dissolved in deionized water (10 mL) at room temperature, respectively. Then, the aqueous solutions of zinc nitrate were added into aqueous solution of 2-MeIm and the mixture was vigorous stirred for 2 h at room temperature. The resulting white precipitate was collected by repeated centrifugation (8000 rpm) for 30 min, further washed by water and methanol for three times, and dried in an oven at 65 °C overnight. 

### 2.3. Preparation of ZIF-L/Epoxy Composites

The composites blended ZIF-L are named as ZIF-L/epoxy. The epoxy resins (10 g), amine curing agent D230 (2 g), and a certain amount of ZIF-L was added into a teflon mold for the curing reaction (8 h and 60 °C). A series of ZIF-L/epoxy composites were successful prepared with 0.25 wt% (0.03 g ZIF-L), 0.5 wt% (0.06 g ZIF-L), 1 wt% (0.12 g ZIF-L), 2.5 wt% (0.24 g ZIF-L), 5 wt% (0.48 g ZIF-L). As a control sample, the neat epoxy (only 10 g epoxy and 2 g D230) without ZIF-L was prepared in a similar way.

### 2.4. Materials Characterizations

The X-ray diffraction (XRD) patterns of samples were acquired by an X-ray diffractometer (Smartlab, Rigaku, Japan) using Cu Kα radiation (λ = 0.154 nm) from 5° to 50° (1°/min). The surface chemical state of ZIF-L was investigated by the Fourier transform infrared spectroscopy (FTIR, Nicolet 6700, Boston, MA, USA) in a range of 4000–400 cm^−1^. The transmission electron microscopy (TEM) images of samples were captured by a TEM instrument (JEM1011, Tokyo Metropolitan, Japan) under a 100 kV acceleration voltage. The micrographs of ZIF-L were observed by a microscope (Hitachi S4800, Tokyo, Japan) at high vacuum with 10.00 kV electric tension. The staining experiments of algae resistance and bacteria were conducted on a TCS SP5 biological laser confocal microscope (LEICA, Wetzlar, Germany). The concentration of bacteria and algae were calibrated by a Microplate Reader (SpectraMax 190, ULTRAOSA, Sacramento, CA, USA). The atomic force microscope (AFM) images of ZIF-L were acquired by a scanning probe microscope (SPM-9700, Kyoto, Japan). The porous structure of ZIF-L nanosheets was further characterized by the nitrogen adsorption-desorption isotherm using Autosorb iQ apparatus from Quantachrome Instruments.

### 2.5. Antibacterial Property Test of ZIF-L

Enterobacteria *Escherichia coli* (ATCC 25922) and marine bacteria *Bacillus subtilis* (ATCC6633) were chosen for the antibacterial activity assessment by the zone of inhibition and shaking flask coated plate method. The ZIF-L powder was pressed into a disc (12 mm diameter × 2 mm thick), and each sample was put onto the surface of an agar plate contained bacteria (106 CFU/mL). The *Bacillus subtilis* (used 2216E agar) and the *Escherichia coli* (used the nutrient agar) were incubated at 37 °C for 24 h.

To determine the anti-*bacillus subtilis* activity of the ZIF-L, a certain volume of *Bacillus subtilis* suspensions were added into the sterilized natural seawater to make sure the bacteria concentration was about 105 CFU/mL in a conical flask. Then, the ZIF-L powder (40 mg) was added into the 40 mL above bacteria suspensions and incubated at 37 °C for 24 h. The mixture (0.1 mL) was taken out from the flask and diluted to ten and hundred times with sterilized natural seawater at each end of the incubation period for 0.5, 1, 2, 4 h, respectively. The decimal dilutions (100 μL) were spread on a Petri dish that contained 2216E agar, and then incubated at 37 °C for 24 h. The number of bacteria colonies were counted on each plate. Three parallel experiments were set for each group of experiments.

To determine the anti-*Escherichia coli* activity of the ZIF-L, we repeated the above operation and replaced seawater with sterile 0.01 M phosphate buffer saline water and 2216E agar with nutrient agar. We marked the amount of microbial colonies on the plate without ZIF-L as Nc, and the amount of microbial colonies on the plate with ZIF-L as Ns.

(1) $$\mathrm{Killing}\;\mathrm{rate}=\frac{\mathrm{Nc}-\mathrm{Ns}}{\mathrm{Nc}}\times 100\%$$

### 2.6. Bacteria and Algae Anti-Adhesion Assessment of ZIF-L/Epoxy Composites

The specimens were placed in 24-well plates and seeded with of *Bacillus subtilis* sterilized natural seawater solution (1000 μL, 108 CFU/mL) and incubated at 37 °C for 24 h (120 rpm). The *Bacillus subtilis* adhesion on composite samples surface was stained via Calcein AM/PI kit and observed by fluorescent inverted microscope and SEM. *Phaeodactylum tricornutum*, *Chlorella*, and *Navicula torgutum* were chosen to investigate the anti-algae attachment performance of the ZIF-L/epoxy composites. The samples were put in 24-well plates and added into algae suspension (1 mL, 108 cells per mL). Washed ZIF-L/epoxy composites with sterilized natural seawater after culturing at 22 °C for 24 h. Then the samples were observed by fluorescent inverted microscope and scanning electron microscopy.

## 3. Results and Discussion

### 3.1. Morphology and Structural Evolution of ZIF-L

**Figure 1:**
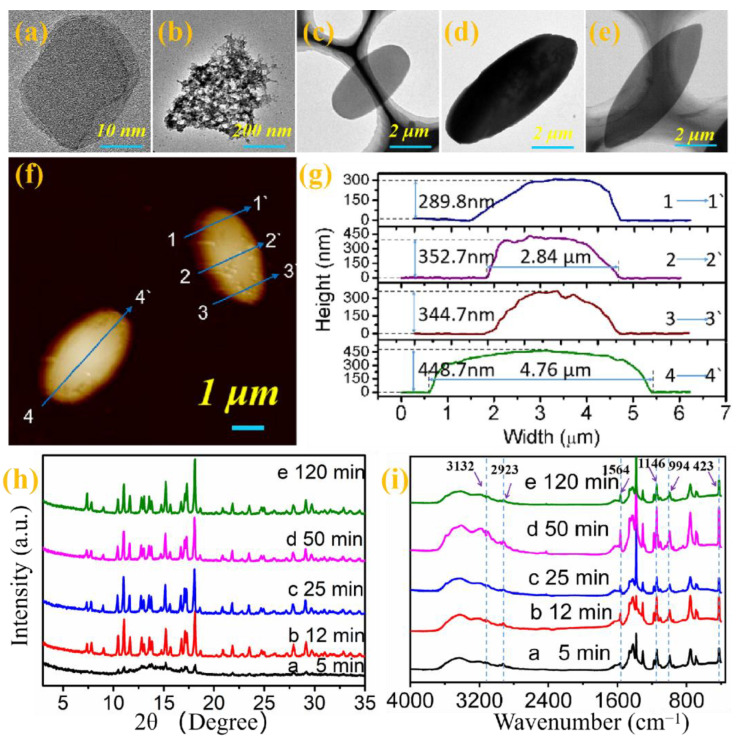
The morphology transformations with the reaction time (**a**) 5 min, (**b**) 12 min, (**c**) 25 min, (**d**) 50 min, (**e**) 120 min, (**f**) SPM image of ZIF-L (**g**) basic parameters of ZIF-L by SPM, (**h**) XRD patterns dependent on the reaction time, (**i**) FT-IR spectrum dependent on the reaction time.

Scheme 1a shows that metal-organic framework (ZIF-L) is synthesized by the coordination of Zn^2+^ and 2-methylimidazole. In this experiment, the morphology transition of ZIF-L dependent on time are investigated by controlling the reaction time at room temperature. As displayed in Figure 1, the morphologies of ZIF-8 nanoparticles are characterized by TEM and SPM. The fine particles are formed from irregular two-dimensional structure (Figure 1a, 5 min), island-like structures (Figure 1b, 12 min), blade-like structures (Figure 1c, 25 min) to leaf-like structures (Figure 1d–f, 50–120 min). As exhibited in the Figure 1f,g, ZIF-L presents a two-dimensional leaf-like structure (height: 448.7 ± 4 nm, length: 4.76 ± 0.2 μm, and width 2.84 ± 0.1 μm). It can be observed that there is no significant difference from XRD peaks of samples at different reaction time (Figure 1h). The characteristic peaks (7.3°, 12.7° and 18.0°) of ZIF-L are derived from (011), (112) and (222) planes [33]. As shown in Figure 1i, it can be seen that the absorption peak at 3132 cm^−1^ is derived from stretching vibration absorption of the C-H bond in the methyl, the peak at 423 cm^−1^ is related to Zn-N stretching. The peak at 2923 cm^−1^ is attributed to imidazole rings, the peak at 1564 cm^−1^ is related to the C=N stretching and the peaks at 1146 and 994 cm^−1^ are from the C-N stretching [34]. The Brunauer–Emmett–Teller surface area and pore volume of the 2D mesoporous ZIF-L nanosheets are calculated to be 38.49 m^2^/g and 0.058 cm^3^/g, respectively, and the average pore size was 6.59 nm as shown in Figure S1.

### 3.2. Antibacterial Activity Evaluation

**Figure 2:**
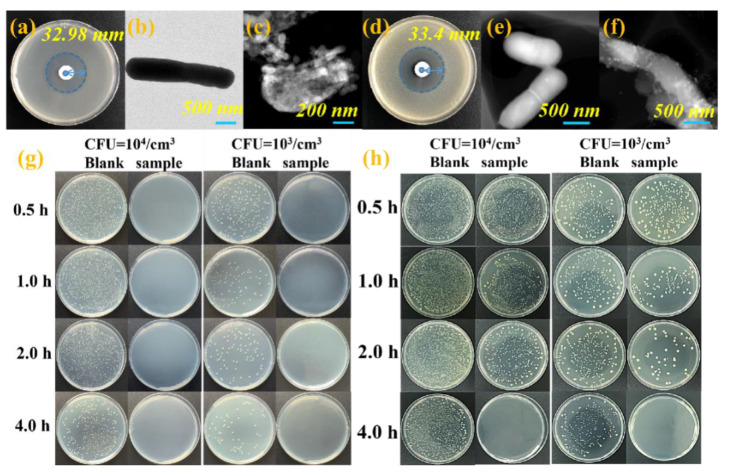
The killing efficiency of the ZIF-L, (**a**) inhibition zone toward *Bacillus subtilis*, (**b**) the TEM of *Bacillus subtilis*, (**c**) the TEM of ZIF-L treated *Bacillus subtilis*, (**d**) inhibition zone toward *Escherichia coli*, (**e**) TEM of *Escherichia coli*., (**f**) TEM of ZIF-L treated *Escherichia coli*, (**g**) photographs of colonies toward *Bacillus subtilis*, (**h**) photographs of colonies toward *Escherichia coli*.

The agar well diffusion method [35], MIC test, and standard plate count method are conducted for exploring the antibacterial activity of ZIF-L. As shown in Figure 2a,d, the culture medium containing *Bacillus subtilis* and *Escherichia coli* both form a zone of inhibition. The inhibition zone diameters of ZIF-L against *Bacillus subtilis* and *Escherichia coli* were 32.98 ± 0.2 mm and 33.40 ± 0.1 mm, respectively, indicating the ZIF-L samples have obvious bactericidal effect. The antimicrobial substrate placed on pathogenic bacteria produce obvious inhibition zone. Because ZIF-L can be degraded in an aquatic environment, and the strong diffusion of Zn^2+^ from ZIF-L on the plate has an impact on the survival and growth of bacteria. The MIC values of ZIF-L to *Bacillus subtilis* and *Escherichia coli* are both 0.5 mg/mL (Figure S2).

**Figure 3:**
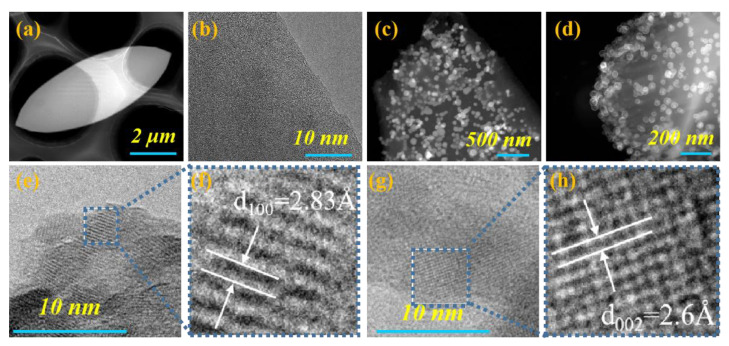
TEM image of the (**a**,**b**) ZIF-L, (**c**) ZIF-L after resistance to *Bacillus subtilis*, (**d**) ZIF-L after resistance to *Escherichia coli* (**e**,**f**) lattice stripes of ZIF-L after resistance to *Bacillus subtilis*, (**g**,**h**) lattice stripes of ZIF-L after resistance to *Escherichia coli*.

The bacterial killing efficiency of ZIF-L is assessed by counting the colonies of bacteria in solution [36] before and after ZIF-L treatment. As exhibited in Figure 2g, no bacterial colonies appear on the petri dishes with the initial concentration (1 mg/mL) of 10^3^ CFU/mL and 10^4^ CFU/mL. It is shown that *Bacillus subtilis* is killed exposure to ZIF-L within half-hour. The sterilization rate of ZIF-L against *Bacillus subtilis* is 100% all the times (Figure S3a,b). The control medium has no toxic effect on *Escherichia coli*, which is consistent with *Bacillus subtilis*. But it can observed that the cytotoxic effect of ZIF-L on *Escherichia coli* is dependent on the time (Figure 2h). When the initial bacterial concentration is 10^4^ CFU/mL, the corresponding sterilization rates are 26.96% (0.5 h), 50.40% (1 h), 86.14% (2 h) and 100% (4 h), respectively (Figure 3c). The corresponding sterilization rates are 20.80%, 27.01%, 83.92%, and 100% when the initial bacterial concentration is 10^3^ CFU/mL (Figure 3d). The bactericidal performance of ZIF-L for Gram-positive is better than that of Gram-negative bacteria [37,38] because the cell wall of gram-positive bacteria is thick, involving the peptidoglycan, teichoic acid and lipoteichoic acid [39]. On the contrary, the gram-negative bacteria has a thin layer of lipopolysaccharide and peptidoglycan as well as an outer membrane on its surface, which can effectively reduce the toxic effect on bacterial cells from reactive oxygen species.

### 3.3. Exploring the Bactericidal Mechanism of ZIF-L

Compared with the smooth and complete rod-like shape from original *Bacillus subtilis* (Figure 2b), the *Bacillus subtilis* treated with ZIF-L cells show serious deformation and even cracks (Figure 2c), implying that ZIF-L can destroy the cell membranes and result in the cytoplasm leakage of *Bacillus subtilis*. The *Escherichia coli* morphology significantly (Figure 2e) after touching ZIF-L in comparison with original morphology (Figure 2f), revealing the same antibacterial mechanism of ZIF-L against Gram-positive and Gram-negative bacteria. Because ZIF-L has strong interactions with bacterial based on the high pressure on the cell of bacterial, puncturing cells, it eventually causes cell deformation and apoptosis [40]. Compared with the original, the leaf shape of ZIF-L (Figure 3a–b) with C, N, Zn elements on the surface (Figure S4a), the morphologies of ZIF-L after being subjected to *Bacillus subtilis* (Figure 3c) and *Escherichia coli* (Figure 3d) have become incomplete, resulting in the new elements (O and P elements) (Figure S4b,b1,c,c1). The appeared element on the surface of ZIF-L may come from *Bacillus subtilis* and *Escherichia coli*. The surface elements of original *Bacillus subtilis* and *Escherichia coli* have been scanned for, verifying the conjecture. There is a large amount of C, N, O elements and a small amount of P element. However, the Zn element almost could not be detected on the surface of *Bacillus subtilis* and *Escherichia coli* (Figure S4c,c1,f,f1). As shown in Figure 2c,f, the *Bacillus subtilis* and *Escherichia coli* no longer have complete bacterial morphologies after touching ZIF-L. The surface of bacteria after treated with ZIF-L contains a large amount of Zn elements and much more P elements in comparison with the original *Bacillus subtilis* and *Escherichia coli* (Figure S4d,d1,g,g1). When ZIF-L is exposed to bacterial solution, Zn^2+^ will be released into the environment. The cell membranes of *Bacillus subtilis* and *Escherichia coli* have negative charges, which can be firmly enriched by Zn^2+^ by coulomb force, finally causing the death of *Bacillus subtilis*. Meanwhile, the imidazole group existing in the ZIF-L is also a widely used bactericidal ingredient to effectively damage the cell membrane of the bacterium [41]. The surfaces of ZIF-L after being subjected to *Bacillus subtilis* and *Escherichia coli* have new zinc oxide nanoparticles. As measured by TEM (high-resolution images), crystal plane spacing of lattice fringes are 2.83 Å (derived from d(100)) and 2.6 Å (002 crystal plane, Figure 3f,h), respectively, indicting Zn^2+^ released by ZIF-L will combine with oxygen in the form of zinc oxide.

### 3.4. Antibacterial Effect of ZIF-L/Epoxy Composites

**Figure 4:**
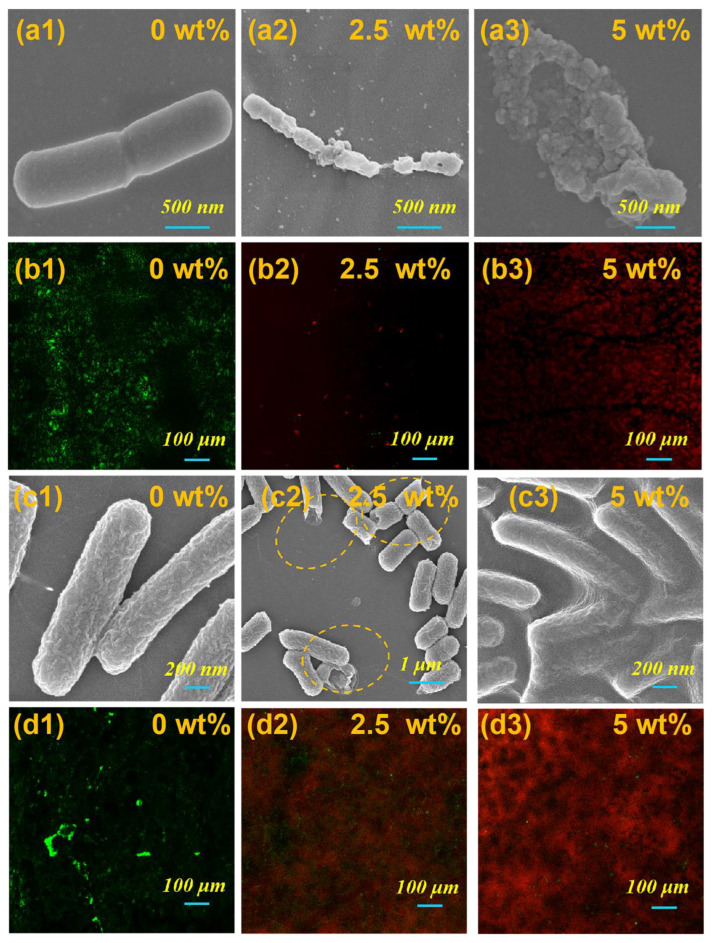
The SEM morphology ((**a1**): 0 wt%, (**a2**): 2.5 wt%, (**a3**): 5 wt%) and live/dead cell staining ((**b1**): 0 wt%, (**b2**): 2.5 wt%, (**b3**): 5 wt%) of *Bacillus subtilis* after touching ZIF-L/epoxy composites with different contents of ZIF-L; The SEM morphology ((**c1**): 0 wt%, (**c2**): 2.5 wt%, (**c3**): 5 wt%) and live/dead cell staining ((**d1**): 0 wt%, (**d2**): 2.5 wt%, (**d3**): 5 wt%) of *Escherichia coli* after touching ZIF-L/epoxy composites with different contents of ZIF-L.

Based on the excellent bactericidal effect of ZIF-L, the bactericidal performances and antibacterial attachement properties of ZIF-L/epoxy are investigated by scanning electron microscopy and live/dead cell staining techniques (notes: red fluorescence spots stand for dead cells and green fluorescence spots represent live cell). *Bacillus subtilis* and *Escherichia coli* on the surface of pure epoxy (0 wt%) exhibit a complete bacterial contour with a slightly wrinkled appearance (Figure 4(a1,c1)). A lot of green fluorescence can be observed under specific wavelength fluorescence with only a few red fluorescent spots (Figure 4(b1,d1)), proving that the pure epoxy resin has no toxic effect on *Bacillus subtilis* and *Escherichia coli*. A large amount of *Escherichia coli* is attached to the surface of pure epoxy resin (Figure S5a). ZIF-L/epoxy composites have good performance in preventing the adhesion of *Escherichia coli* (Figure S5b,c,d,e). It is worth noting that the removal rate (*Escherichia coli*) for ZIF/epoxy composites can be reached to 90.20% by only adding ZIF-L (0.25 wt%). The *Bacillus subtilis* and *Escherichia coli* on the surface of the ZIF-L (2.5 wt%)/epoxy composite shows incomplete morphology with the internal substance of the bacteria leaking out (Figure 4(a2) and circled in Figure 4(c2)). As seen in Figure 4(a3,c3), the overall bacteria morphologies on the surface of ZIF-L (5 wt%)/epoxy became distorted with a large amount of internal substances leaking from the bacteria in vitro, indicting the obvious toxic effect on *Bacillus subtilis Escherichia coli* from ZIF-L (5 wt%)/epoxy composites. (Figure 4(b3,d3)).

### 3.5. Algae Resistance of ZIF-L/Epoxy Composites

**Figure 5:**
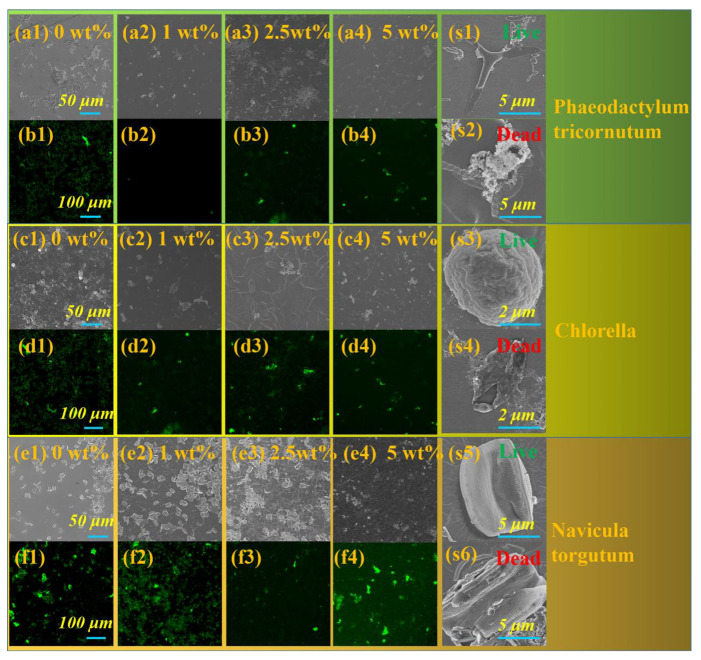
The SEM morphology ((**a1**): 0 wt%, (**a2**): 1 wt%, (**a3**): 2.5 wt%, (**a4**): 5 wt%, (**s1**,**2**)) and fluorescence image ((**b1**): 0 wt%, (**b2**): 1 wt%, (**b3**): 2.5 wt%, (**b4**): 5 wt%) of *Phaeodactylum tricornutum* after touching ZIF-L/epoxy composites with different contents of ZIF-L; The SEM morphology ((**c1**): 0 wt%, (**c2**): 1 wt%, (**c3**): 2.5 wt%, (**c4**): 5 wt%, (**s3**,**4**)) and fluorescence image ((**d1**): 0 wt%, (**d2**): 1 wt%, (**d3**): 2.5 wt%, (**d4**): 5 wt%) of *Chlorella* after touching ZIF-L/epoxy composites with different contents of ZIF-L; The SEM morphology ((**e1**): 0 wt%, (**e2**): 1 wt%, (**e3**): 2.5 wt%, (**e4**): 5 wt%, (**s5**,**6**)) and fluorescence image ((**f1**): 0 wt%, (**f2**): 1 wt%, (**f3**): 2.5 wt%, (**f4**): 5 wt%) of *Navicula torgutum* after touching ZIF-L/epoxy composites with different contents of ZIF-L.

As selected three algae *phaeodactylum tricornutum*, *chlorella*, and *navicula torgutum* as the research objects, the algae resistance performance of ZIF-L/epoxy composites is investigated based on the broad-spectrum antifouling ability. The anti-algae properties of ZIF-L/epoxy composite can be explored by using fluorescence microscope technology and scanning electron microscope (notes: the chlorophyll contained in the plant can be excited by the fluorescence). As shown in Figure 5(a1,c1,e1), three kinds of algae can be observed attached to a large area on the surface of pure epoxy resin. As shown in Figure 5(a2–a4), the attachment rates of *Phaeodactylum tricornutum* for ZIF/epoxy is only 0.13% (1 wt% ZIF), 0.89% (2.5 wt% ZIF) and 0.87% (5 wt% ZIF), respectively, in comparison to that of *phaeodactylum tricornutum* for pure epoxy resin (12.94%). Moreover, as displayed in Figure 5(c2–c4), the attachment rates of *Chlorella* for ZIF/epoxy are only 0.20% (1 wt% ZIF), 1.33% (2.5 wt% ZIF) and 0.36% (5 wt% ZIF) respectively, in comparison with that of *Chlorella* for pure epoxy resin (7.37%, Figure 5(c1)). Meanwhile, as displayed in Figure 5(e2–e4), the attachment rates of *Navicula torgutum* for ZIF/epoxy are only 10.31% (1 wt% ZIF), 6.83% (2.5 wt% ZIF) and 2.19% (5 wt% ZIF), respectively, in comparison with that of *phaeodactylum tricornutum* for pure epoxy resin (11.42%, Figure 5(e1)).

The measurement of fluorescence tests shows that pure epoxy matrix have no effect on algae in terms of green fluorescence (no leakage of chlorophyll, Figure 5(b1,d1,e1)). Compared with pure epoxy, the ZIF/epoxy composites have excellent algae-resistant properties (incomplete cell structure, Figure 5(b2–b4, d2–d4 and f2–f4)). Figure 5s exhibits morphologies of the algae (live/dead) by SEM. It is observed that the algae (*Phaeodactylum tricornutum*, *Chlorella* and *Navicula torgutum*) exposed to ZIF/epoxy composites have an incomplete cell structure with the organelles in the body leaking out (Figure 5(s2,s4,s6)). From the above observation, ZIF-L/epoxy composites can efficiently kill gram-negative bacteria, gram-positive bacteria, and algae, preventing the formation of biofilm and fundamentally avoid biofouling.

## 4. Conclusions

The metal-organic frame material (ZIF-L) is synthesized with a 2-dimensional leaf-like morphology by simple aqueous solution synthesis. ZIF-L have highly antibacterial activity in simulated physiological environments and the marine environment. The ZIF-L nanosheet exhibits complete inactivation for *Escherichia coli* (PBS: 4 h) and *Bacillus subtilis* (seawater: 0.5 h). The ZIF-L/epoxy composite not only has excellent antibacterial effect, but also a poisoning effect and antifouling effect on a variety of marine algae. It is worth noting that the removal rate of *Escherichia coli* for ZIF-L/epoxy composite can reach 90.20% by only adding 0.25 wt% of ZIF-L. This work will inspire researchers to develop advanced metal-organic materials in the antibacterial and anti-algae fields.

## Data Availability

The data presented in this study are available upon request from the corresponding authors.

## Supplementary Materials

The following supporting information can be downloaded at https://www.mdpi.com/article/10.3390/nano13010202/s1, Figure S1: N_2_ adsorption-desorption isotherms and its pore-size distribution curve (inset) of ZIF-L; Figure S2: The images showing MIC measurement results; Figure S3: (a) the elimination ratio of the ZIF-L toward *Bacillus subtilis* (CFU=10^4^/cm^3^), (b) the elimination ratio of the ZIF-L toward *Bacillus subtilis* (CFU=10^3^/cm^3^), (c) the elimination ratio of the ZIF-L toward *Escherichia coli* (CFU=10^4^/cm^3^), (d) the elimination ratio of the ZIF-L toward *Escherichia coli* (CFU=10^4^/cm^3^).; Figure S4: Surface element analysis of the (a) ZIF-L, (b, b1) ZIF-L after resistance to *Bacillus subtilis*, (c, c1) *Bacillus subtilis*, (d, d1) *Bacillus subtilis* after antibacterial treatment.(e, e1) ZIF-L after resistance to *Escherichia coli*, (f, f1) *Escherichia coli*, (g, g1) *Escherichia coli* after antibacterial treatment; Figure S5: The SEM morphology (a: 0 wt%, b: 0.25 wt%, c: 1 wt%) and live/dead cell staining (d: 0.25 wt%, e: 1 wt%) of *Escherichia coli* after touching ZIF-L/epoxy composites with different contents of ZIF-L.
