# Supplementary material for: Preparation of 2D ZIF-L and Its Antibacterial and Antifouling Properties
Source: Nanomaterials (Basel). 2023 Jan 2;13(1):202. doi: 10.3390/nano13010202 (PMC9824576; doi:10.3390/nano13010202)
Supplement: Supplementary file 1 [file nanomaterials-13-00202-s001.zip › nanomaterials-2077723-supplementary.pdf]

Preparation of 2D ZIF-L and Its Antibacterial and Antifouling
Properties

Jingyu Li [1,2], Yang Zhang [1,2], Haichao Zhao [3,*] and Guoxin Sui [1,2,*]

1 Shi-Changxu Innovation Center for Advanced Materials, Institute of Metal Research, Chinese Academy of Sciences, Shenyang 110016, China
2 School of Materials Science and Engineering, University of Science and Technology of China, Shenyang 110016, China
3 Key Laboratory of Marine Materials and Related Technologies, Zhejiang Key Laboratory of Marine Materials and Protective Technologies, Ningbo Institute of Materials Technology and Engineering, Chinese Academy of Sciences, Ningbo 315201, China
* Correspondence: zhaohaichao@nimte.ac.cn (H.Z.); gxsui@imr.ac.cn (G.S.)

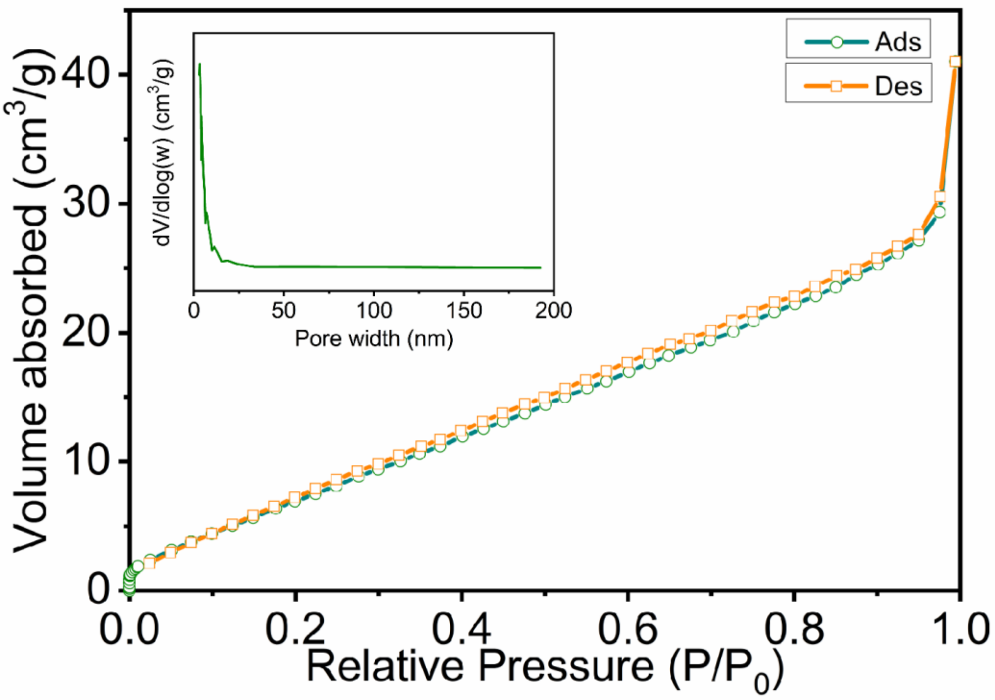

Figure S1: N_2 adsorption-desorption isotherms and its pore-size distribution curve (inset) of ZIF-L.

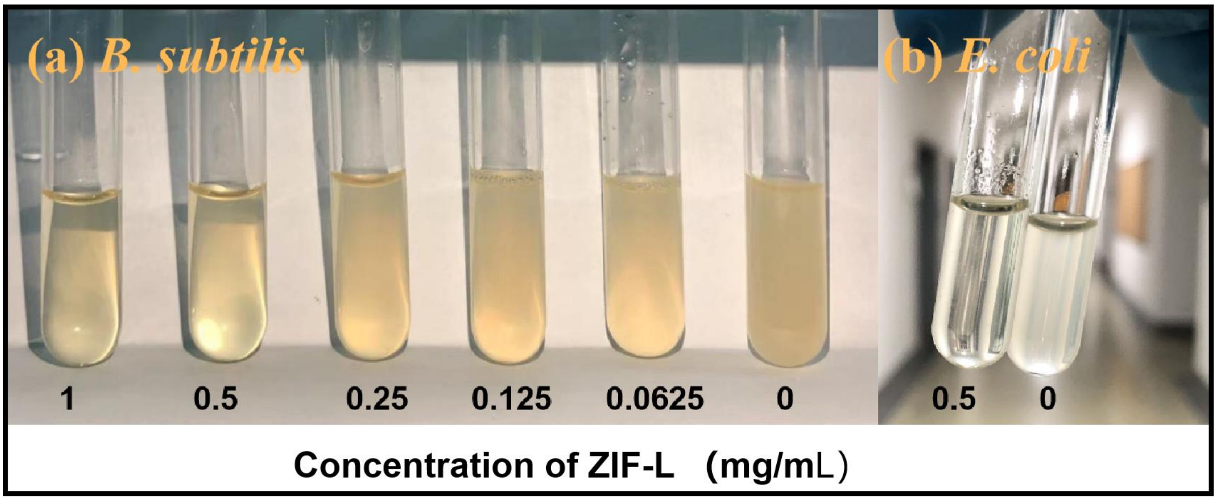

Figure S2： The images showing MIC measurement results.

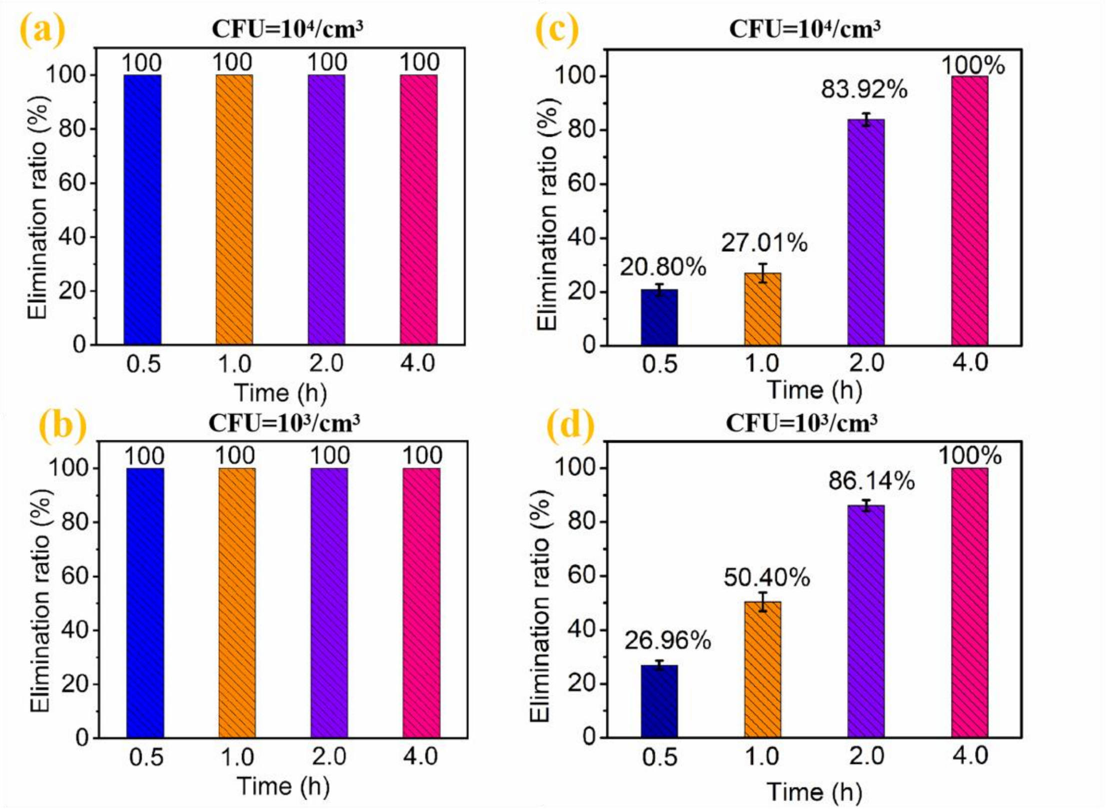

Figure S3: (a) the elimination ratio of the ZIF-L toward Bacillus subtilis (CFU=10^4/cm^3), (b) the elimination ratio of the ZIF-L toward Bacillus subtilis (CFU=10^3/cm^3), (c) the elimination ratio of the ZIF-L toward Escherichia coli (CFU=10^4/cm^3), (d) the elimination ratio of the ZIF-L toward Escherichia coli (CFU=10^4/cm^3).

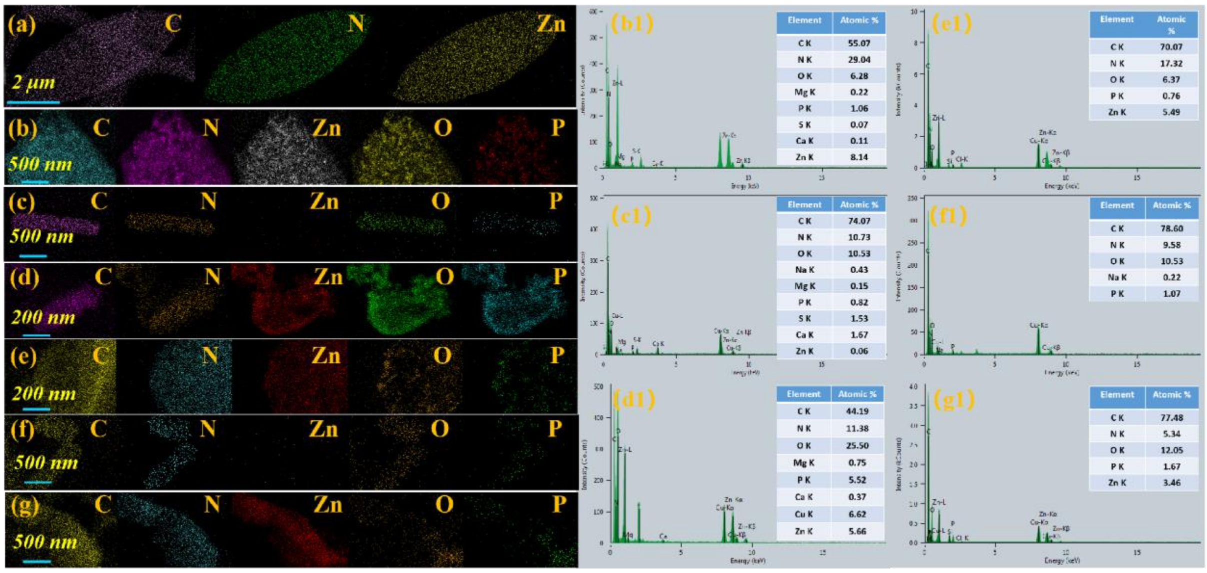

Figure S4： Surface element analysis of the (a) ZIF-L, (b, b1) ZIF-L after resistance to *Bacillus subtilis*, (c, c1) *Bacillus subtilis*, (d, d1) *Bacillus subtilis* after antibacterial treatment.(e, e1) ZIF-L after resistance to *Escherichia coli*, (f, f1) *Escherichia coli*, (g, g1) *Escherichia coli* after antibacterial treatment

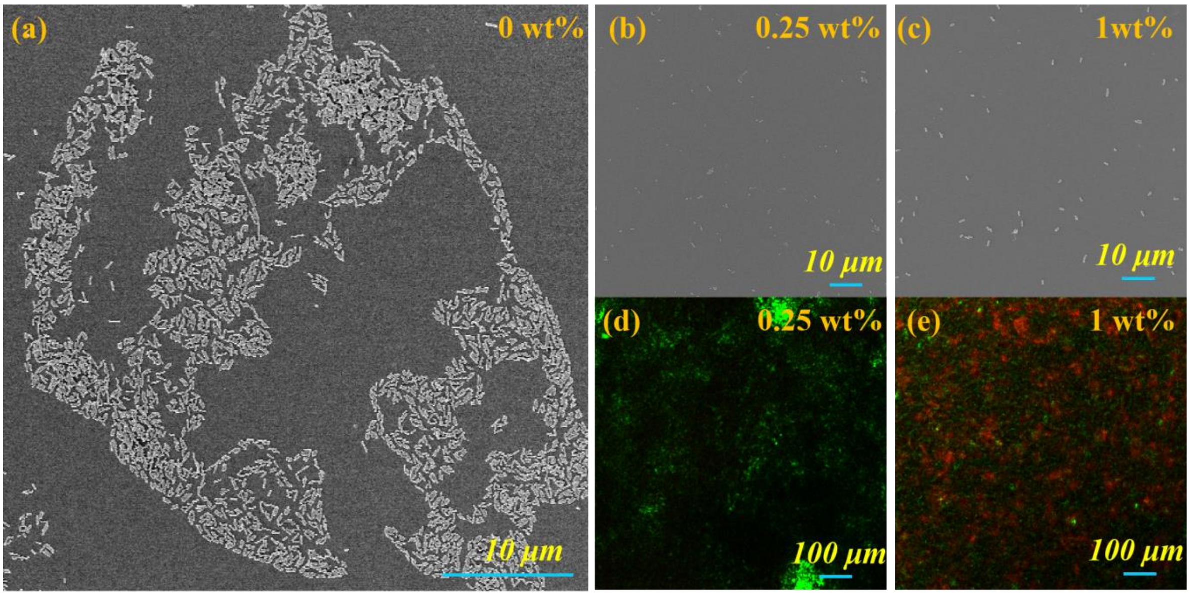

Figure S5： The SEM morphology (**a**: 0 wt%, **b**: 0.25 wt%, **c**: 1 wt%) and live/dead cell staining (**d**: 0.25 wt%, **e**: 1 wt%) of *Escherichia coli* after touching ZIF-L/epoxy composites with different contents of ZIF-L.
